# How Social Preferences Shape Incentives in (Experimental) Markets for Credence Goods[Link]

**DOI:** 10.1111/ecoj.12284

**Published:** 2016-02-23

**Authors:** Rudolf Kerschbamer, Matthias Sutter, Uwe Dulleck

**Affiliations:** ^1^ University of Innsbruck; ^2^ University of Cologne and University of Innsbruck; ^3^ Queensland University of Technology

## Abstract

Credence goods markets suffer from inefficiencies caused by superior information of sellers about the surplus‐maximising quality. While standard theory predicts that equal mark‐up prices solve the credence goods problem if customers can verify the quality received, experimental evidence indicates the opposite. We identify a lack of robustness with respect to heterogeneity in social preferences as a possible cause of this and conduct new experiments that allow for parsimonious identification of sellers’ social preference types. Our results confirm the assumed heterogeneity in social preferences and provide strong support for our explanation of the failure of verifiability to increase efficiency.

A central topic in the field of information economics is the design of institutions or contracts that mitigate market inefficiencies resulting from the presence of asymmetric information. Almost all contributions to the literature build on the assumption of common knowledge that agents are rational own‐money maximisers who behave as desired when kept indifferent in own‐money terms – see Bolton and Dewatripont (2005) for a textbook coverage of this approach. In this article, we argue that while this assumption is harmless in some applications – because it results in institutions that are almost optimal if preferences are almost as assumed – it is misleading in others.

Specifically, we study markets for credence goods where inefficiencies result from superior information of sellers about the optimal quality for consumers. In such markets, theory predicts that equal‐mark‐up prices solve the problem if customers can verify the quality received (Dulleck and Kerschbamer, 2006). However, this prediction is refuted by existing experimental evidence which indicates that markets with verifiability perform no better than markets without (Dulleck *et al*., 2011). We identify a lack of robustness of institutional design with respect to heterogeneity in social preferences as a possible cause. By social preferences, we mean that subjects may not only care for their own material pay‐off but may consider the pay‐offs of others as well, when making decisions.

To provide support for our explanation for the failure of verifiability to increase efficiency, we design a simple and intuitive test that allows for parsimonious identification of a seller's social preference type. The results obtained in an implementation of the test indicate that less than a fourth of the experimental sellers behave in accordance with the standard assumption on preferences. The rest behave either in line with other forms of selfish preferences or in accordance with different variants of non‐selfish social preferences. Taken together our experimental findings provide strong support for heterogeneity in social preferences and for our explanation of the failure of verifiability to increase efficiency. Based on this observation, we argue that future research should search for an institutional design that is robust against preference heterogeneity. Such research seems especially important for markets for credence goods where inefficient institutions potentially cause huge economic costs.^1^

The next subsection describes the main problems emerging from the asymmetric information in markets for credence goods and explains how verifiability helps to solve them in theory. The subsequent subsection summarises the experimental evidence showing that verifiability fails empirically and sketches our explanation for the failure.

## 0.1 Credence Goods Markets, Informational Asymmetries and the Role of Verifiability

Credence goods markets are characterised by informational asymmetries between expert sellers and customers because customers are unable to identify the quality they need, whereas expert sellers are able to do so (Darby and Karni, 1973). Typical examples include:

- health care services, where the doctor is better informed than the patient on the disease the latter has and on the treatment he needs;
- car repair services, where the mechanic knows more about the type of service the vehicle needs than the owner; and
- taxicab rides in an unknown city, where the driver is better informed about the shortest route to the destination than the tourist.

A second informational problem in markets for credence goods arises when the customer is unable to observe and verify the quality of service he has received. For example, in the market for medical treatments a patient might be unable to distinguish a cheap from an expensive drug infusion. In the car repair market, the owner might be unable to observe whether a broken part has been repaired or replaced.

The informational asymmetries on credence goods markets may cause a variety of problems and inefficiencies. Expert sellers may provide unnecessarily high quality (a case referred to as ‘overtreatment’), or insufficiently low quality (‘undertreatment’), or they may charge for a higher quality than provided (‘overcharging’). Such cases are not only a theoretical possibility but are well documented in the literature. Empirical evidence for considerable market inefficiencies is available, among others, for the health care sector (Hughes and Yule, 1992; Gruber and Owings, 1996; Gruber *et al*., 1999; Iizuka, 2007), for car repairs (Wolinsky, 1993; Hubbard, 1998; Schneider, 2012) and for taxi rides (Balafoutas *et al*., 2013).

An important finding in the theoretical literature is that verifiability ensures efficiency on markets for credence goods.^2^ Verifiability applies if consumers are able to observe and verify the quality they receive, so that expert sellers cannot charge for a quality that has not been provided. If verifiability applies, experts are predicted to choose equal‐mark‐up prices. With such prices an expert earns the same profit independently of the quality she provides (sellers are referred to as she and consumers as he throughout to make the reference clear although in practice they can both be of either sex). Thus, under the mentioned standard assumption on preferences, such prices induce the expert to provide the appropriate quality of the credence good. As a consequence, consumers – inferring experts’ incentives from posted prices – are predicted to interact and the market is predicted to reach the maximal level of efficiency.

## 0.2 The Limits of Verifiability and a Potential Explanation for Its Failure

Experimental data in Dulleck *et al*. (2011) indicate that – contrary to theoretical prediction – verifiability fails to promote efficiency on credence goods markets. Indeed, the relative frequencies of market interaction, undertreatment and overtreatment do not differ significantly between two experimental treatments that are identical except that verifiability applies to one but not the other. The observed aggregate performance in both treatments is better in terms of efficiency than the standard prediction for a market without verifiability but considerably worse than the prediction for a market with verifiability. These findings raise two questions whose answers are important for the understanding of – and the optimal design of institutions for – credence goods markets: why is the performance of credence goods markets so poor in the presence of verifiability when all theoretical approaches predict verifiability to ensure efficiency? And why do markets without verifiability perform so much better than predicted?

In this article, we argue that heterogeneity in the social preferences of credence goods sellers can provide an answer to both questions. Key to our argument are the following two observations:

- the standard solution to the credence goods problem for the case where the quality of the good is verifiable – equal‐mark‐up prices – is robust against the presence of sellers with pro‐social other‐regarding preferences but non‐robust against the presence of sellers with anti‐social other‐regarding concerns. By pro‐social (anti‐social) other‐regarding preferences we mean a willingness to give up own material pay‐off to increase (decrease) the material pay‐off of the trading partner;
- for the prediction for markets without verifiability the opposite is true – it is robust against the presence of sellers with anti‐social other‐regarding preferences but non‐robust against the presence of sellers with pro‐social other‐regarding concerns.

A key ingredient in our explanation in the previous paragraph is heterogeneity in social preferences in the (experimental) seller population. To provide support for heterogeneity, we design new experiments intended to identify a seller's social preferences from her provision behaviour. Our main theoretical innovation is the construction of a simple and intuitive test that allows us to identify a seller's social preference type without making any specific assumptions on the form of her utility function. This distinguishes our approach from most of the rest of the literature on elicitation of type and intensity of social preferences, which uses test designs that rely on strong assumptions regarding the form of the utility function.^3^ ${}^{,}$ 4

We then implement our test for social preferences in new credence goods markets experiments. Our main findings are that:

- only a minority (of less than a quarter) of subjects behave according to the standard assumption of lexicographic maximisation of first the own and then the other's material pay‐off;
- the behaviour of a sizeable minority of subjects is consistent with other forms of selfish preferences;
- the behaviour of a large majority of sellers is consistent with either a taste for efficiency (in the spirit of Andreoni and Miller (2002) or Charness and Rabin (2002)) or inequality aversion (in the tradition of Fehr and Schmidt (1999) or Bolton and Ockenfels (2000)); and
- a minority of subjects behaves spitefully or competitive (*à la* Levine (1998) or Charness and Rabin (2002)).

Hence, our empirical findings provide strong support for heterogeneity in social preferences and therewith for our explanation for the surprisingly low level of efficiency on credence goods markets in the presence of – and the surprisingly high efficiency level in the absence of – verifiability.

The remainder of the article is organised as follows. Section 1 first introduces a simple model of a credence goods market, then presents predictions based on standard assumptions and finally reports the results from two experimental treatments in Dulleck *et al*. (2011). Section 2 presents our explanation for the low level of efficiency in credence goods markets in the presence and the high level of efficiency in the absence of verifiability in the data of Dulleck *et al*. (2011). Section 3 develops the test for identifying social preferences in a credence goods experiment and Section 4 presents the results from an implementation of the test. Section 5 concludes with a discussion of our results and their implications for institutional design and for agent selection.

## 1 Verifiability in Credence Goods Markets: Model, Standard Predictions and Experimental Evidence

### 1.1 Basic Model

Consumers are *ex ante* identical. They need a high quality, $q^{1}$, of a particular (credence) good with probability *h*, and a low quality, $q^{0}$, with probability 1 − *h*. Each consumer (he) is randomly matched with one seller (she) who sets prices $p^{1}$ and $p^{0}$ for the high, respectively low, quality (with $p^{1}\;\geq \;p^{0}$). The seller has costs $c^{1}$ ($c^{0}$, respectively) for the high (low) quality, with $c^{1}\;>\;c^{0}$.

The consumer only knows the prices for the different qualities but not the quality he needs when he makes his decision whether or not to interact with the seller. In case of interaction, the seller gets to know which quality the customer needs. Then, she provides one of the two qualities and charges one of the two prices.

Customers in need of the low quality are sufficiently treated in either case, both if the seller chooses $q^{0}$ and if she chooses $q^{1}$. However, if the customer needs the high quality, then only $q^{1}$ is sufficient. A sufficient quality yields a value *v* > 0 for the customer, an insufficient quality yields a value of zero. If the customer decides against interaction then both, the customer and the seller, receive an outside option of *o* ≥ 0. In case of an interaction, the monetary pay‐off for the consumer is the value from the quality received minus the price to be paid. The seller receives the monetary pay‐off of the price charged minus the costs of the quality provided. More formally, let *θ* ∈ {0,1} be the index of a customer's need in terms of quality, *μ* ∈ {0,1} the index of the quality provided and *κ* ∈ {0,1} the index of the quality charged for. Then the material pay‐off of the seller under price‐vector $\left(p^{0},p^{1}\right)$ is

(1) $$\pi_{s}\left(p^{0},p^{1},\mu ,\kappa \right)=p^{\kappa }-c^{\mu },$$

while the customer receives

(2) $$\pi_{c}\left(p^{0},p^{1},\theta ,\mu ,\kappa \right)=v-p^{\kappa },\;\text{if}\;\theta \leq \mu \text{, and}\;-p^{\kappa }\;\text{otherwise.}$$

**Figure 1:**
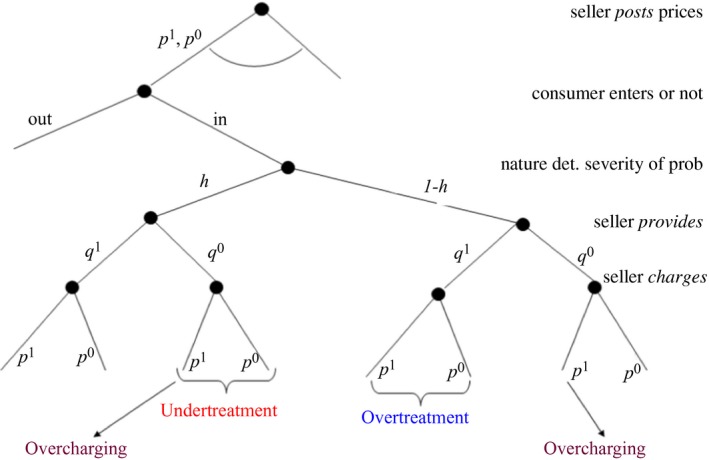
The Credence Goods Game *Notes*. The term undertreatment refers to providing $q^{0}$ when the consumer needs $q^{1}$; overtreatment refers to providing $q^{1}$ when the consumer needs $q^{0}$; and overcharging refers to charging $p^{1}$ when $q^{0}$ has been provided.

Figure 1 presents this game. Note that this simple game captures all the idiosyncratic problems of credence goods markets discussed in the introduction. If a customer needs $q^{1}$ and the seller provides $q^{0}$, we have undertreatment; if the customer needs $q^{0}$ and the seller provides $q^{1},$ we have overtreatment; and if the seller charges $p^{1}$ when $q^{0}$ is provided, we have overcharging.

### 1.2 Experimental Design

In the following, we introduce the experimental parameterisation of the basic model used in Dulleck *et al*. (2011) which are also used in our new experiments below.^5^ We refer to two treatments in Dulleck *et al*. (2011), one without verifiability (treatment N‐Endo) and one with verifiability (treatment V‐Endo).^6^ Treatment N‐Endo corresponds to the game shown in Figure 1. Implementing verifiability means that consumers are able to observe and verify *ex post* the quality of the provided good (without knowing, however, whether this quality is the appropriate one). Therefore, in treatment V‐Endo the last stage in Figure 1 is degenerate because the expert has to charge the price for the provided quality. Hence, with verifiability overcharging is precluded, while over‐ and undertreatment are still possible.

In both treatments the customer's probability of needing the high quality is *h* = 0.5, and the value of a sufficient quality is *v* = 10. The costs of providing the low (high) quality are $c^{0}\;=\;2$ ($c^{1}\;=\;6$). The prices posted by the sellers, $p^{0}$ and $p^{1}$ (with $p^{0}\;\leq \;p^{1}$), have to be chosen in integer numbers from the interval {1, … , 11}. The outside option if no trade takes place between the seller and the customer is set to *o* = 1.6.

Matching groups of eight subjects each were implemented, with four subjects as customers and four subjects as sellers. Role assignment was random at the beginning and fixed for all 16 periods in the experiment. In order to prevent attempts to build up a reputation as a reliable seller, there was random rematching of customers and sellers within each matching group after each period. All experimental sessions were run computerised using zTree (Fischbacher, 2007) and recruiting was done via ORSEE (Greiner, 2004). A total of 184 subjects participated in treatments N‐Endo and V‐Endo.

### 1.3 Standard Prediction for the Role of Verifiability

Prediction 1 (Standard Prediction for the Role of Verifiability). Under the assumption that subjects have standard preferences, in treatment N‐Endo no interaction will take place, yielding no efficiency gains in the market. By contrast, in treatment V‐Endo the expert will post $p^{0}\;=\;6$ and $p^{1}\;=\;10$ and the consumer will choose to enter the market and he will get the appropriate quality, yielding full efficiency in the market.

The following considerations lead to this prediction. Consider treatment N‐Endo first. Under the standard assumption of common knowledge that all agents are rational, risk‐neutral and exclusively interested in their own material pay‐off, the expert will always charge the higher price $p^{1}$ and always provide the cheaper quality $q^{0}$. Anticipating this, a consumer will then only accept if $p^{1}\;\leq \;\left(1\;-\;h\right)v\;-\;o\;=\;3.4$. But with such a $p^{1}$ the seller earns less than the value of her outside option (because $\left(1\;-\;h\right)v\;-\;c^{0}\;<\;2o$). Thus, no interaction is predicted for N‐Endo. In treatment V‐Endo, the expert cannot charge for a quality other than the provided one and the quality provided depends on the mark‐up $p^{\mu }\;-\;c^{\mu },$ *μ* ∈ {0,1}. An equal‐mark‐up price‐vector is defined as one that satisfies $p^{1}\;-\;c^{1}\;=\;p^{0}\;-\;c^{0}$. Under the standard assumption on preferences (that if indifferent in own‐money terms the expert will provide in the best interest of the customer) an equal‐mark‐up price‐vector is predicted to induce provision of appropriate quality. An undertreatment (overtreatment) price‐vector satisfies $p^{1}\;-\;c^{1}\;<\;p^{0}\;-\;c^{0}$ ($p^{1}\;-\;c^{1}\;>\;p^{0}\;-\;c^{0}$) and is predicted to induce provision of low (high) quality independently of the customer's need.

**Figure 2:**
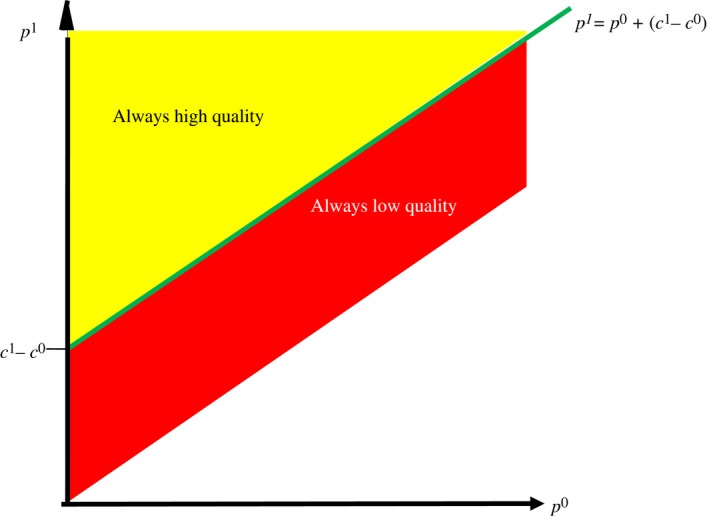
Standard Prediction for Provision Behaviour under Verifiability *Notes*. Under the standard assumption on preferences an expert's provision behaviour under verifiability is determined exclusively by her material incentives: if $c^{1}\;-\;c^{0}\;>\;p^{1}\;-\;p^{0}$ ($c^{1}\;-\;c^{0}\;<\;p^{1}\;-\;p^{0}$) the expert earns more by selling $q^{0}$ ($q^{1}$) and is therefore predicted to always provide the low quality (high quality); if $c^{1}\;-\;c^{0}\;=\;p^{1}\;-\;p^{0}$ the expert is indifferent in material terms and in this case standard theory predicts that she will provide the appropriate quality.

Figure 2 shows in the space of price‐vectors the set of equal‐mark‐up price‐vectors as a straight line with slope 1. The set of undertreatment price‐vectors is indicated as the dark area below the equal‐mark‐up line and the set of overtreatment vectors is shown as the light area above the equal‐mark‐up line. Anticipating how an expert's provision behaviour depends on the price‐vector under which the transaction takes place, a consumer will accept an equal‐mark‐up vector iff $p^{1}\;\leq \;10$, an undertreatment vector iff $p^{0}\;\leq \;3$, and an overtreatment vector iff $p^{1}\;\leq \;8$. Thus, to maximise profits, the expert will post the equal‐mark‐up vector $\left(p^{0},p^{1}\right)\;=\;\left(6,10\right),$ which will be accepted by an own‐money‐maximising, risk‐neutral consumer.

### 1.4 Experimental Results of Dulleck *et al*. (2011)

Observation 1 (Experimental Results for the Role of Verifiability). Compared to treatment N‐Endo, verifiability has no significant impact on the frequency of interaction, the undertreatment rate, the overtreatment rate and overall efficiency. The overall performance in both treatments is better than the standard prediction for treatment N‐Endo but worse than the standard prediction for treatment V‐Endo.

**Figure 3:**
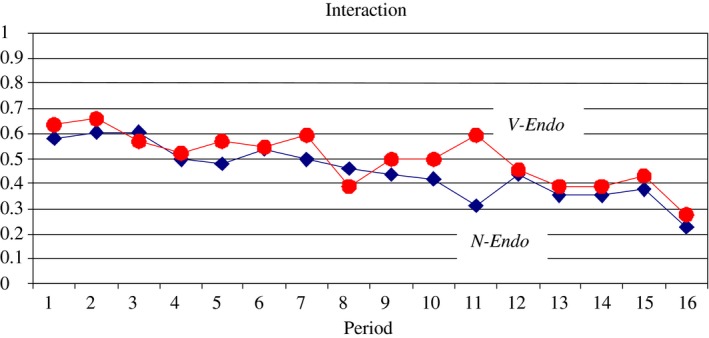
Relative Frequency of Interaction in N‐Endo and V‐Endo *Notes*. The Relative Frequency of Interaction is calculated as (no. accepted transactions)/(no. possible interactions) averaged over all sessions for a given treatment.

**Figure 4:**
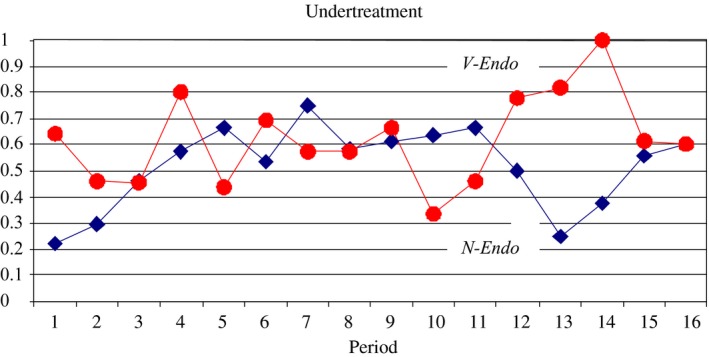
Relative Frequency of Undertreatment in N‐Endo and V‐Endo *Notes*. The Relative Frequency of Undertreatment is calculated as (no. cases where the customer needs $q^{1}$ but receives $q^{0}$)/(no. cases where the customer needs $q^{1}$) averaged over all sessions for a given treatment.

Table 1, as well as Figures 3 and 4 support this observation, leading us to reject both parts of Prediction 1: contrary to the prediction efficiency gains and interaction rates are not significantly different between the two treatments and they are significantly higher than 0 and significantly lower than 1 in both.

**Table 1:** Summary Statistics for N‐Endo and V‐Endo

Averages per period	N‐Endo*	V‐Endo*
Interaction	0.45	0.50
Undertreatment^†^	0.53	0.60
Overtreatment^‡^	0.06	0.05
Overcharging^§^	0.88	–
Profit seller	2.69	2.58
Profit customer	1.00	1.06
Number of subjects	96	88
(independent matching groups)	(12)	(11)

*Notes*. *None of the variables is significantly different between the two treatments (using two‐sided Mann–Whitney U‐tests with matching groups of eight subjects as independent observations). ^†^Customer needs $q^{1}$, but seller provides $q^{0}$. ^‡^Customer needs $q^{0}$ but seller provides $q^{1}$. ^§^Seller provides $q^{0}$ but charges $p^{1}$ (with $p^{1}\;>\;p^{0}$ and customer needs $q^{0}$).

A possible explanation for the relatively high interaction rate and the relatively low undertreatment rate in N‐Endo is experts having a taste for efficiency. Another possible explanation is that experts care for equitable pay‐offs. Support for the latter hypothesis comes from the analysis of price‐posting behaviour. Contrary to the theoretical prediction, equal‐mark‐up prices are very rare in V‐Endo. They are chosen in less than 5% of all transactions. Table 2 reports the frequencies of the five most popular price‐vectors posted by sellers in the two treatments. It is interesting to note that in treatment V‐Endo only one equal‐mark‐up vector is among the top five price‐vectors but it is not the predicted one. In both treatments, the price‐vector (6, 8) is by far the most frequently posted price‐vector. If the seller always provided the appropriate quality and charged for it, then this price‐vector would split the gains from trade equally between the consumer and the seller both when the consumer needs the low and when he needs the high quality. The prominence of this price‐vector therefore suggests that a concern for relative pay‐offs plays a role for aggregate behaviour in the experiment.

**Table 2:** Most Popular Price‐vectors in N‐Endo and V‐Endo

Treatment N‐Endo			Treatment V‐Endo		
$\left(p^{0},p^{1}\right)$	Absolute no.	Relative frequency (%)	$\left(p^{0},p^{1}\right)$	Absolute no.	Relative frequency (%)
(6,8)	176	22.92	(6,8)	265	37.64
(4,8)	84	10.94	(7,8)	89	12.64
(5,7)	50	6.51	(5,8)	46	6.53
(5,8)	44	5.73	(4,8)	17	2.41
(4,7)	39	5.08	(8,8)	15	2.13
	393 (of 768)	51.17		432 (of 704)	61.36

Of course, these observations provide only a rough indication that social preferences may shape sellers’ behaviour. In Section 3, we develop a simple parsimonious test for social preferences within the framework of a credence goods market which is then implemented in new experiments in Section 4. Before doing so we argue (in Section 2) that heterogeneity in social preferences can explain why markets with verifiability perform worse than in the standard prediction and why markets without verifiability perform better.

## 2 Heterogeneity in Social Preferences and Robustness of Institutions

In this Section, we explain in more detail how heterogeneity in social preferences of sellers can explain the relatively bad performance of credence goods markets with verifiability and the relatively good performance of markets without verifiability. Our discussion here and in the next Section relies on the assumption that (experimental) credence goods sellers are heterogeneous and that the preferences of each seller can be represented by a utility or motivation function $U(\pi_{s},\pi_{c})$ satisfying the following three conditions:

- $\partial U/\partial \pi_{s}>0;$
- $\mathrm{sign}(\partial U/\partial \pi_{c})$ depends (only) on whether $\pi_{s}\geq \pi_{c},$ or $\pi_{s}<\pi_{c};$ and
- $\partial U/\partial \pi_{s}>\partial U/\partial \pi_{c}.$

The first condition requires that – holding the monetary pay‐off of the customer constant – the seller's utility increases in her own monetary pay‐off. This assumption is satisfied by all empirically relevant social preference types discussed in the economics literature.

The second assumption states that whether a seller is selfish, pro‐social or anti‐social depends only on whether the customer has more or less monetary pay‐off than the seller. On the one hand, this assumption is generous because it allows for all empirically relevant types of social preferences that have been discussed in the economics literature. On the other hand, this assumption is strong because it implies

- that the seller's general attitude towards the customer only depends on the pay‐off allocation implied by a decision; and
- that the reference point for the evaluation of pay‐off allocations is an outcome with equal pay‐offs for both sides of the market.^7^

The third assumption states that a seller values changes in own material pay‐off more than equivalent or smaller changes in the customer's pay‐off. This assumption is fairly innocent for allocations with $\pi_{s}\;<\;\pi_{c}$ but might be regarded as somewhat restrictive for allocations with $\pi_{s}>\pi_{c};$ its main purpose is to get a unique ‘switching point’ in the test proposed below, though, and it can be relaxed without changing results qualitatively.^8^

Given our three assumptions on the utility or motivational function $U(\pi_{s},\pi_{c})$, it seems natural to distinguish between the five archetypes of social preferences defined in Table 3.^9^ What can we say about the market behaviour of credence goods sellers exhibiting those types of social concerns?

**Table 3:** Social Preference Types and Implied Provision Behaviour

Social preference type	Derivative of *U* w.r.t. $\pi_{c}$		Provision behaviour under Ω	
	For $\pi_{s}\geq \pi_{c}$	For $\pi_{s}\;<\;\pi_{c}$	Cust. needs $q^{0}$	Cust. needs $q^{1}$
SE (selfish)	=0	=0	$q^{0}$ or $q^{1}$	$q^{0}$ or $q^{1}$
EL (efficiency loving)	>0	>0	$q^{0}$	$q^{1}$
SP (spiteful)	<0	<0	$q^{1}$	$q^{0}$
IA (inequality averse)	>0	<0	$q^{1}$	$q^{1}$
IL (inequality loving)	<0	>0	$q^{0}$	$q^{0}$

Consider markets without verifiability (*N*‐markets) first. For such markets the standard prediction – undertreatment and overcharging under each price‐vector – is already a worst case scenario that leaves no room for deterioration. This follows from the observation that by behaving according to the standard prediction a seller not only maximises her material pay‐off but also minimises the pay‐off of the customer. An immediate consequence is that anti‐social other‐regarding preferences do not manifest themselves in a worse outcome than predicted under standard preferences. On the other hand, pro‐social other‐regarding preferences easily manifest themselves in a better market outcome than predicted. To see this, consider an EL expert who finds out that the customer needs $q^{1}$. By providing $q^{0}$ instead of $q^{1}$ she increases her material pay‐off by $c^{1}\;-\;c^{0}$ at a cost of $v\;>\;c^{1}\;-\;c^{0}$ to the customer. Thus, if the additional profit the seller receives from providing $q^{0}$ instead of $q^{1}$ (i.e. $c^{1}\;-\;c^{0}$) is small compared to the loss arising from undertreatment (i.e. *v*) and if the weight on $\pi_{c}$ in her utility function is sufficiently high relative to the weight on $\pi_{s}$, she will refrain from undertreatment. The same is true for IA experts in the domain of advantageous inequality and for IL experts in the domain of disadvantageous inequality.

In sum, in *N*‐markets experts with anti‐social other‐regarding preferences behave exactly like experts with standard preferences while experts with pro‐social other‐regarding preferences tend to behave better than predicted by standard theory.

**Figure 5:**
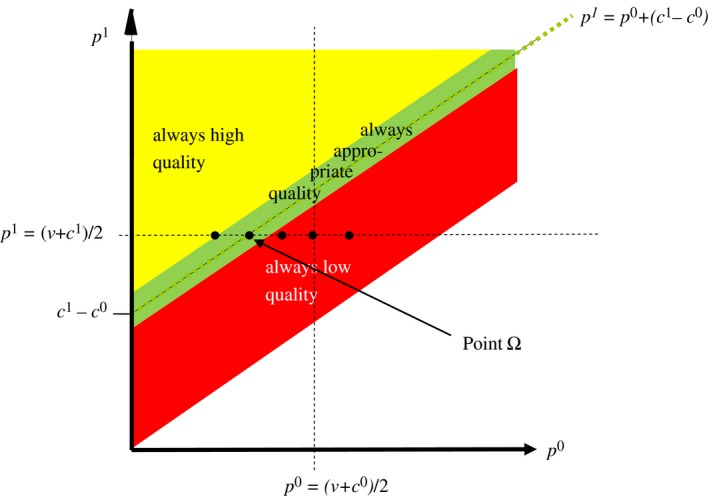
Provision Behaviour of an EL Expert Under Verifiability *Notes*. An EL expert is willing to give up own material pay‐off to increase the material pay‐off of the customer. Thus, she will necessarily provide the appropriate quality in a corridor along the equal‐mark‐up line (where helping the customer involves no cost).

For the standard solution for markets with verifiability (*V*‐markets), by contrast, we get the opposite result. To see this, note that the standard prediction for equal‐mark‐up prices – appropriate quality independent of the level of the mark‐up – is already a best‐case scenario that leaves no room for improvement. Consider an EL expert, for instance. Since the material pay‐off of the customer enters positively in her utility function, she will act in the interest of the consumer along the equal‐mark‐up line, where helping the customer involves no cost. Furthermore, since $\partial U/\partial \pi_{c}\;>\;0$ in both domains (i.e. in the domain of advantageous inequality and in the domain of disadvantageous inequality) the EL expert will provide the appropriate quality even under price‐vectors that deviate (slightly) from the equal‐mark‐up rule. Thus, EL experts necessarily provide appropriate quality in a corridor along the equal‐mark‐up line – as shown in Figure 5 – but they do not perform better than SE experts at the equal‐mark‐up line.^10^ The same is true for other experts with pro‐social other‐regarding preferences – under equal‐mark‐up prices they behave as predicted but do not behave better than predicted. However, anti‐social other‐regarding preferences easily manifest themselves in a worse market outcome than predicted under standard preferences because hurting the customer involves no cost under equal‐mark‐up prices. Consider a SP expert, for instance. Since the material pay‐off of the customer enters negatively in her utility function, she necessarily provides $q^{1}$ to a consumer who needs $q^{0},$ and $q^{0}$ to a consumer who needs $q^{1}$, along the equal‐mark‐up line where hurting the customer involves no cost. Furthermore, since $\partial U/\partial \pi_{c}\;<\;0$ in both domains the SP expert will always provide the wrong quality even under price‐vectors that deviate (slightly) from the equal‐mark‐up rule. The same is true for other experts with negative attitudes towards customers – most importantly for IA experts in the domain of disadvantageous inequality.

Together these observations not only explain the poor performance of equal‐mark‐up prices in V‐Endo, they also explain why equal‐mark‐up prices are very rarely chosen in this treatment. More importantly, there is no cheap repair for this problem in the sense that there is simply no price‐vector that induces a SP expert, for instance, to provide the appropriate quality in a V‐market. Her provision behaviour is rather (qualitatively) like the one shown in Figure 5 with the important difference that she will necessarily always provide the wrong (instead of the appropriate) quality in a corridor along the equal‐mark‐up line.

## 3 Identification of Social Preferences in Markets for Credence Goods

The discussion in the previous section assumes that there is heterogeneity in social preferences in the (experimental) expert population. The challenge is, of course, to show that empirically. Based on the three primitive assumptions on preferences introduced in the previous section we now derive a parsimonious test for the identification of social preferences in the framework of a credence goods market with verifiability. Our starting point in deriving the test is the following observation: in the space of possible price‐vectors there is exactly one (and only one) that allows for a neat discrimination between the above defined preference types from the provision behaviour in a credence goods market. Looking at Figure 5 it is the price‐vector referred to as ‘Point Ω’. It is defined as follows:

Definition 1 The price‐vector $\Omega \;=\;(p_{\Omega }^{0},\;p_{\Omega }^{1})$ has $p_{\Omega }^{0}\;=\;\left(v\;+\;c^{1}\right)/2\;-\;\left(c^{1}\;-\;c^{0}\right)$ and $p_{\Omega }^{1}\;=\;\left(v\;+\;c^{1}\right)/2$.

To discuss the properties of this price‐vector, we have first to define and discuss the location of the three dashed lines in Figure 5:

- The upward sloping dashed line is the equal‐mark‐up line. It connects all price‐vectors with $p^{1}\;-\;p^{0}\;=\;c^{1}\;-\;c^{0}$, implying that the expert receives exactly the same material pay‐off independently of whether she provides $q^{0}$ or $q^{1}$ at points on this line.
- The horizontal dashed line connects all price‐vectors where the expert and the customer receive exactly the same material pay‐off if the expert (correctly or incorrectly) provides $q^{1}$. Thus, this line is defined by $\pi_{s}\left(p^{0},\;p^{1},\;\mu \;=\;1,\;\kappa \;=\;1\right)\;=\;\pi_{c}\left(p^{0},\;p^{1},\;\theta \;=\;1,\;\mu \;=\;1,\;\kappa \;=\;1\right)\;=\;\pi_{c}\left(p^{0},\;p^{1},\;\theta \;=\;0,\;\mu \;=\;1,\;\kappa \;=\;1\right)⟺p^{1}\;=\;\left(v\;+\;c^{1}\right)/2.$
- The vertical dashed line connects all price‐vectors where the expert and the customer receive exactly the same material pay‐off if the expert correctly provides $q^{0}$. Thus, this line is defined by $\pi_{s}\left(p^{0},\;p^{1},\;\mu \;=\;0,\;\kappa \;=\;0\right)\;=\;\pi_{c}\left(p^{0},\;p^{1},\;\theta \;=\;0,\;\mu \;=\;0,\;\kappa \;=\;0\right)⟺p^{0}\;=\;\left(v\;+\;c^{0}\right)/2.$

Since Point Ω is at the intersection of the upward sloping and the horizontal dashed line it has $p^{0}\;=\;\left(v\;+\;c^{0}\right)/2\;-\;\left(c^{1}\;-\;c^{0}\right)/2,$ implying that this point is necessarily to the left of the vertical dashed line – where we have $p^{0}\;=\;\left(v\;+\;c^{0}\right)/2$.

Now, suppose we (as the experimentalists) impose the price‐vector in Point Ω and look at an expert's provision behaviour. First, assume the customer needs the cheaper quality, $q^{0}$. If the expert provides the appropriate quality, she induces a pay‐off allocation $\left(\pi_{s},\pi_{c}\right)$ with disadvantageous inequality. This is so because Point Ω is strictly to the left of the vertical dashed line along which both parties get exactly the same material pay‐off if the expert correctly provides $q^{0}$. If the expert provides the expensive quality instead, she induces an equal‐material‐pay‐offs allocation – that is, an allocation with $\pi_{s}\;=\;\pi_{c}$. This follows from the fact that Point Ω is on the horizontal dashed line. Furthermore, since Point Ω is on the equal‐mark‐up line, the expert's own material pay‐off is the same in both allocations.

What does this imply for provision behaviour? An EL expert and an IL expert will necessarily decide for the asymmetric allocation – by providing $q^{0}$ to a customer who needs $q^{0}$. By contrast, an SP and an IL expert necessarily decide for the symmetric allocation – by providing $q^{1}$ to a customer who needs $q^{0}$. This is so because the own material pay‐off is the same in both allocations, while the customer's pay‐off is higher in the asymmetric than in the symmetric allocation (relevant for EL and SP) respectively because disadvantageous inequality is present in the asymmetric but absent in the symmetric allocation (relevant for IA and IL).

Now assume that the customer needs the expensive quality, $q^{1}$. If the expert provides $q^{1}$, then she induces the equal‐material‐pay‐offs allocation discussed in the previous paragraph. This follows from the fact that the material pay‐off of both parties is independent of the quality needed by the customer when the expensive quality is provided. If the expert provides $q^{0}$ instead, she induces a pay‐off allocation $\left(\pi_{s},\;\pi_{c}\right)$ with advantageous inequality. This follows from the fact that Point Ω has $p^{0}\;=\;\left(v\;+\;c^{0}\right)/2\;-\;\left(c^{1}\;-\;c^{0}\right)/2$ which exceeds $c^{0}/2$ because $v\;>\;(c^{1}\;-\;c^{0})$. Furthermore, since Point Ω is on the equal‐mark‐up line, the expert's own material pay‐off is the same in both allocations.

From these considerations it follows that an EL expert and an IA expert will necessarily decide for the symmetric allocation – by providing $q^{1}$ to a customer who needs $q^{1}$ – while a SP and an IL expert necessarily decide for the asymmetric allocation – by providing $q^{0}$ to a customer who needs $q^{1}$. This is so because the own material pay‐off is the same in both allocations while the customer's pay‐off is higher in the symmetric than in the asymmetric allocation (relevant for EL and SP) respectively because advantageous inequality is present in the asymmetric but absent in the symmetric allocation (relevant for IA and IL).

In sum, if we observe the decision of an expert under the price‐vector located at Point Ω in Figure 5 twice, once combined with the consumer needing the low quality and once combined with the consumer needing the high quality, then we can infer her social preference type with some precision – see Table 3. To formulate a more precise statement, we call the strategy of providing the appropriate quality in both cases ‘always appropriate quality’ and the strategy of providing the expensive quality in both cases ‘always high quality’; moreover, we denote the strategy of providing the cheap quality in both cases ‘always low quality’ and the strategy of providing the expensive quality when the cheap quality is needed and the cheap quality when the expensive one is needed ‘always wrong quality’. Using those terms, we can state the following Proposition:

Proposition 1 (Impartial Social Preferences). Consider the price‐vector Ω as defined in Definition 1. Under this price‐vector:

- ‘always appropriate quality’ is consistent with SE and EL preferences but inconsistent with IA, SP and IL;
- ‘always high quality’ is consistent with SE and IA preferences but inconsistent with EL, SP and IL;
- ‘always low quality’ is consistent with SE and SP preferences but inconsistent with IA, SP and EL;
- ‘always wrong quality’ is consistent with SE and SP preferences but inconsistent with IA, EL and IL.

Follows immediately from the text preceding the result.

Testing the provision behaviour under the price‐vector Ω is like eliciting impartial social preferences, because under this price‐vector a seller compares two allocations that yield the same material pay‐off for her but different pay‐offs for the customer. Thus, deciding for the ‘fair’ allocation (whatever is considered fair) does not involve any costs here. Based on the predictions for Point Ω we now change $p^{0}$ slightly, while keeping $p^{1}$ constant, in order to test whether (experimental) sellers are willing to give up their own material pay‐off to help or hurt the customer. From Figure 5, an increase (decrease) in $p^{0}$ corresponds to a move along the horizontal dashed line to the right (left) of Point Ω implying that we increase (decrease) the expert's pay‐off from providing $q^{0}$ at the cost (for the benefit) of the consumer's pay‐off. At the same time, the pay‐offs for both parties from providing $q^{1}$ remain constant at the equal‐material‐pay‐offs allocation $\left(\pi_{s},\;\pi_{c}\right)\;=\;\left[\left(v\;-\;c^{1}\right)/2,\;\left(v\;-\;c^{1}\right)/2\right].$

Given our three assumptions on the utility function, what are the implications of changing $p^{0}$ for the provision behaviour of sellers with different types of social preferences? First, we get the following monotonicity result:

Lemma 1 (Monotonicity). Consider two price‐vectors, the price‐vector Ω from Definition 1 and a second vector, Ψ, which has the same $p^{1}$ as Ω (i.e. $p_{\Psi }^{1}\;=\;p_{\Omega }^{1}$) but a different $p^{0}$ (i.e. $p_{\Omega }^{0}\;\neq \;p_{\Psi }^{0}$). If $p_{\Omega }^{0}\;<\;p_{\Psi }^{0}$ ($p_{\Omega }^{0}\;>\;p_{\Psi }^{0}$ respectively) then – keeping the consumer's need with respect to quality constant – an expert who provides $q^{0}$ ($q^{1}$) under Ω must provide $q^{0}$ ($q^{1}$)under Ψ.

See online Appendix B.

Proposition 1 and Lemma 1 together imply:

Proposition 2 (Partial Social Preferences). Consider the price‐vectors Ω and Ψ from Lemma 1. Then observing

- ‘always appropriate quality’ under Ω and Ψ is only consistent with EL preferences (but inconsistent with SE, IA, SP and IL);
- ‘always high quality’ under Ω and ‘always high quality’, ‘always appropriate quality’ or ‘always wrong quality’ under Ψ with $p_{\Omega }^{0}\;<\;p_{\Psi }^{0}$ is only consistent with IA preferences (but inconsistent with SE, EL, SP and IL);
- ‘always low quality’ under Ω and ‘always low quality’, ‘always appropriate quality’ or ‘always wrong quality’ under Ψ with $p_{\Omega }^{0}\;>\;p_{\Psi }^{0}$ is only consistent with IL preferences (but inconsistent with SE, IA, SP and EL);
- ‘always wrong quality’ under Ω and ‘always wrong quality’ under Ψ is only consistent with SP preferences (but inconsistent with SE, IA, EL and IL).

To understand Proposition 2, the test to be applied in the next Section, and the term ‘partial social preferences’, consider an IA seller, for instance. From the arguments above, we know that such an expert will always provide the high quality under price‐vector Ω. Increasing $p^{0}$ slightly, while keeping $p^{1}$ constant, creates a tension between a higher own monetary pay‐off and more inequality. By deciding for ‘always high quality’ or switching to ‘always appropriate quality’ (or ‘always wrong quality’) the seller reveals a positive willingness to pay for reducing inequality, because own‐money‐maximisation would ask for ‘always low quality’. The argument for sellers with other kinds of social preferences is similar.

## 4 Implementing the Test in Lab Experiments

### 4.1 Experimental Parameters and Procedures

**Figure 6:**
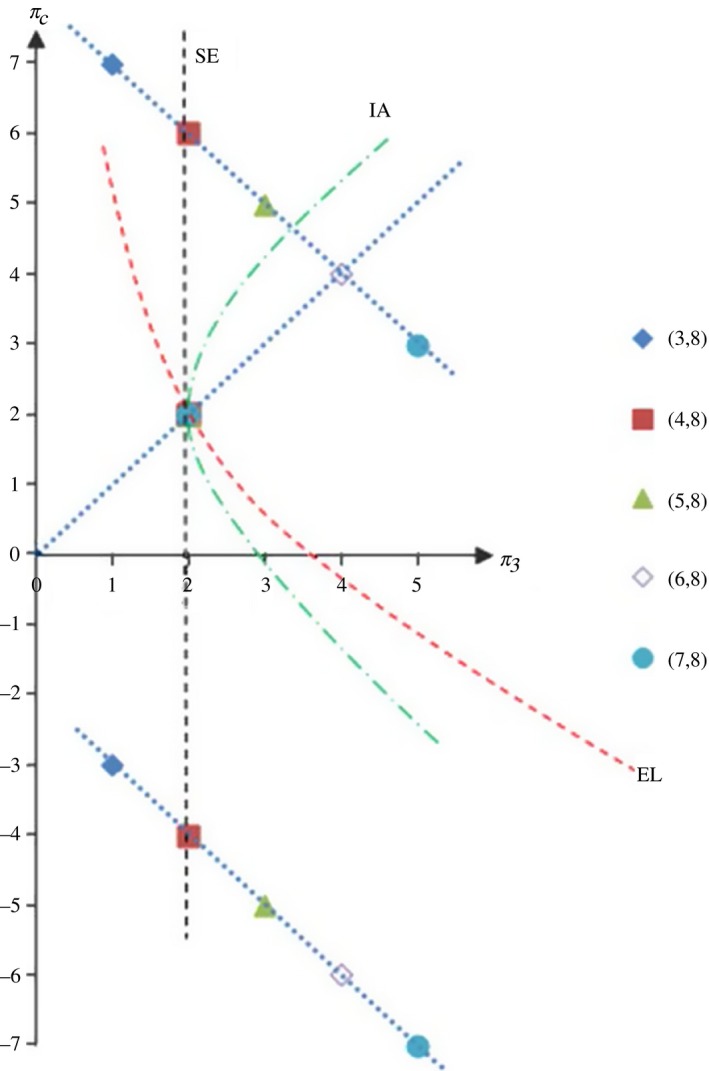
Possible Combinations of Buyer's and Seller's Material Pay‐offs (for different price‐vectors and depending on whether the buyer needs $q^{0}$ or $q^{1}$) *Notes*. Providing $q^{1}$ induces the equal‐material‐pay‐offs outcome $\left(\pi_{s},\pi_{c}\right)\;=\;\left(2,2\right)$ independently of the needed quality under each of the considered price‐vectors. If the customer needs $q^{0}$, the seller implicitly chooses between this allocation (by inefficiently providing $q^{1}$) and the allocation corresponding to the respective price‐vector on the line (with slope −1) above the equal‐material‐pay‐offs allocation (by efficiently providing $q^{0}$). If the customer needs $q^{1}$ the choice is between the equal‐material‐pay‐offs point (by efficiently providing $q^{1}$) and the respective point on the line (with slope −1) below the equal‐material‐pay‐offs allocation (by inefficiently providing $q^{0}$).

To test for and classify the social preferences of sellers, we ran new experiments using a design based on the theoretical results derived in the previous Section. The timing of the game was exactly the same as in the game described in Section 1, except for the first stage: instead of letting sellers post their prices themselves, the price‐vector in a given period was chosen exogenously – through the software – with equal probability from the set {(3,8), (4,8), (5,8), (6,8), (7,8)}. This set of vectors has two characteristics:

First and foremost, it includes the equal‐mark‐up vector Ω characterised in Proposition 1 – it is the vector (4,8). Starting from this price‐vector it then varies $p^{0}$ as described in Lemma 1 and Proposition 2. The allocations implied by the equal‐mark‐up vector Ω and by the other price‐vectors in the set are displayed in Figure 6.

Second, this set of price‐vectors includes the four most frequently chosen price‐vectors in treatment V‐Endo (see Table 2). We call the experimental treatment with this (exogenously given) set of price‐vectors V‐Exo1. In order to check whether the inclusion of the price‐vector (3,8) – which was not among the most frequently posted price‐vectors in treatment V‐Endo – has any impact on behaviour, we also ran an experimental treatment where the exogenously determined price‐vector was chosen with equal probability only from the four most frequently chosen price‐vectors (4,8), (5,8), (6,8) and (7,8). We call this treatment V‐Exo2.

We ran four sessions with 16 subjects each both for V‐Exo1 and for V‐Exo2, yielding eight independent matching groups per treatment. Overall, a total of 128 subjects participated in the new experiments (with no subject having participated in the experiment reported in Section 1). Sessions lasted less than 1.5 hours.

### 4.2 Experimental Results

Tables 4 and 5 present the data for the two new treatments with exogenously imposed price‐vectors, that is, for V‐Exo1 and for V‐Exo2. From Table 4, it is clear that – except for overtreatment – there are no significant differences between those treatments (using the overall average for a particular variable within a matching group of eight subjects as the independent observation, yielding eight independent observations per treatment). For overtreatment, the Table indicates significantly higher rates in V‐Exo1 than in V‐Exo2. A closer look at the data reveals that the difference is entirely due to the provision behaviour under the price‐vector (3,8), which is present in V‐Exo1 but absent in V‐Exo2. In fact, Table 5 shows that – controlling for price‐vectors – there is no significant difference between V‐Exo1 and V‐Exo2, both with respect to overtreatment and undertreatment. This also implies that, conditional on the price‐vector and the quality needed by the consumer, there is no difference in the likelihood of appropriate treatment across treatments.^11^ These results allow us to pool the data from the two treatments in the following analysis of social preference types.

**Table 4:** Overview of Results in V‐Exo1 and V‐Exo2 (periods 7–16)

	V‐Exo1	V‐Exo2	p‐value
Interaction	0.54	0.58	0.40
Undertreatment	0.53	0.46	0.71
Overtreatment	0.35	0.22	0.05*
Profit seller	2.27	2.35	0.40
Profit buyer	1.29	1.29	0.60
Number of subjects	64	64	
(independent matching groups)	(8)	(8)	

*Notes*. *However, we do not find a significant difference in overtreatment between V‐Exo1 and V‐Exo2 if we control for the price‐vector (see Table 5): the provision behaviour under the price‐vector (3,8) – which is present in V‐Exo1 but absent in V‐Exo2 – seems to be responsible for the difference in overtreatment between V‐Exo1 and V‐Exo2.

**Table 5:** Undertreatment (UT) and Overtreatment (OT) Rates Conditional on Price‐vectors (periods 7–16)

$p_{I},p_{II}$	*UT* ^*^	*UT* ^*^	p‐value	*OT* ^†^	*OT* ^†^	p‐value
	V‐Exo1	V‐Exo2	(*UT*)^*^	V‐Exo1	V‐Exo2	(*OT*)^†^
(3,8)	0.048	n.a.	–	0.913	n.a.	–
(4,8)	0.100	0.243	0.07	0.333	0.529	0.17
(5,8)	0.737	0.778	1.00	0.000	0.100	0.24
(6,8)	0.882	0.765	0.23	0.077	0.000	0.28
(7,8)	0.818	0.636	0.41	0.000	0.000	n.a.

*Notes*. *Undertreatment: customer needs $q^{1}$, but seller provides $q^{0}$. ^†^Overtreatment: customer needs $q^{0}$, but seller provides $q^{1}$.

In order to classify sellers according to their social preferences, we first look at violations of monotonicity according to Lemma 1. It turns out that 45 out of 64 sellers (70%) behave in line with the statement over all 16 periods of the experiment. Taking into account that some learning may go on in early periods, we decided to focus on the final 12 periods only (i.e. on periods 5–16). In those periods the behaviour of 56 out of 64 sellers (88%) respects the monotonicity condition. This high degree of consistent behaviour is encouraging, because it suggests that stable (non‐standard) preferences, rather than noise or any kind of confusion of subjects, drives our findings. Of the 56 sellers whose behaviour is consistent with Lemma 1, we had to exclude three from further analysis due to lack of data caused by customers’ opting out.^12^ Our data analysis is therefore based on 53 sellers.

Observation 2 (Identification of Social Preferences).

- Less than a quarter of the experimental sellers act in accordance with the standard assumption on preferences – they provide appropriate quality if and only if they are held indifferent in own‐money terms;
- About a quarter of the seller population displays behaviour that is consistent with a strong taste for efficiency. They provide appropriate quality even if own‐money maximisation calls for over‐ or undertreatment;
-  About a fifth of sellers show behaviour that is consistent with strong inequality aversion. They over or under‐treat customers if this behaviour reduces inequality (or turns disadvantageous into advantageous inequality) even if it also reduces their own monetary pay‐off;
- Adding up strong and weak forms of social preferences indicates that about half of the sellers display behaviour that is consistent with a taste for efficiency, while little more than a quarter of the sellers display behaviour consistent with (strong or weak) inequality aversion.

Table 6 provides a summary of the data.^13^ To read it properly, note that sellers who are classified as either weak EL, weak IA, weak SP or weak IL are also classified as weak SE. This has to be the case because weak EL, IA, SP and IL behave exactly as the strong version of the respective type as ‘impartial spectators’ (i.e. when there is no trade‐off between own material pay‐off and a fairness standard), that is at price‐vector (4,8) in Figure 6. Once $p^{0}$ varies, weak EL, IA, SP and IL act exactly like (strong) SE, because their own material pay‐off is at stake.^14^ It follows that for relative frequencies (given in parentheses in Table 6) to add up to 100%, one has to add up either the strong non‐SE types and the total number of SE types or the total number of non‐SE types and the number of strong SE types.

**Table 6:** Classification of Individual Behaviour in V‐Exo

Social preference type	Strong	Weak	Total
EL (efficiency loving)	13 (24.5%)	13 (24.5%)	26 (49.0%)
IA (inequality averse)	10 (18.9%)	3 (5.7%)	13 (24.5%)
SP (spiteful)	0 (0%)	3 (5.7%)	3 (5.7%)
IL (inequality loving)	0 (0%)	2 (3.8%)	2 (3.8%)
*SE* (selfish)	9 (17.0%)	21 (39.6%)	30 (56.6%)

*Notes*. Note that sellers who are classified as either weak EL, weak IA, weak SP or weak IL are also classified as weak SE. Thus, for relative frequencies (given in parentheses) to add up to 100%, one has to add up either the strong non‐SE types and the total number of SE types or the total number of non‐SE types and the number of strong SE types.

It is important to recall that the results displayed in Table 6 allow for ‘mistakes’ in early rounds. If we do not allow for learning in early periods then we lose eight of the 53 observations. Interestingly, we do not lose a single experimental seller who reveals a willingness to give up own material pay‐off to change the material pay‐off of the customer.^15^ This suggests that selfish sellers do need some time to find out the own‐money‐maximising strategy while strong EL and strong IA ‘know how they want to behave’ from the beginning. Since strong EL and strong IA reveal a willingness to give up own material pay‐off to change the material pay‐off of the customer, while the other types appearing with non‐zero entries in Table 6 do not, one would expect that the former two types earn less – on average – than the rest. This is indeed what we find in the data. Tables C4 and C5 in the online Appendix C display – for the seller types in Table 6 – the average profits per period conditional on an interaction having taken place. While the entries for strong EL and strong IA are 2.37 and 2.22 respectively the other types earn 2.48, on average. If we put strong EL and strong IA sellers in one tub and all the other sellers in a second tub then the difference across tubs in average profits per period (conditional on an interaction having taken place) is significant at the 5% level (p = 0.03, Mann‐Whitney U‐test, *N* = 53).

### 4.3 Discussion of Heterogeneity of Preferences

An important insight from our experimental results is that the behaviour of only a minority of individuals (those in the category ‘weak EL’) is consistent with the standard assumption on preferences – that is that sellers always follow their monetary incentives and in case of indifference they act in the interest of customers. This insight is important for several reasons. First, it is important for the current application – institutional design for credence goods markets under verifiability – because it provides an explanation for both, why equal‐mark‐up price‐vectors do not work as predicted by theory and why such vectors were not chosen in the endogenous pricing conditions of Dulleck *et al*. (2011). Second, it is important for institutional design for markets plagued by asymmetric information more generally, because it suggests that institutional design based on the standard assumption on preferences might yield bad incentives for some or even many agents. The results reported in Table 6 also confirm the heterogeneity in social preferences on which our discussion in Section 2 is based. Some sellers care for efficiency, some for equality of pay‐offs, and some do not care for the well‐being of others (or for efficiency) at all.

Heterogeneity in preferences and behaviour is a well‐established finding, of course. Indeed, it has been observed in many other games, for instance in public goods games (Fischbacher *et al*., 2001; Fischbacher and Gächter, 2010) or in gift‐exchange games (Fehr *et al*., 1997, 1993). Also, in the literature on identification of the type and intensity of social preferences, heterogeneity is well known (Andreoni and Miller, 2002; Charness and Rabin, 2002; Engelmann and Strobel, 2004; Fisman *et al*., 2007). The current article contributes to the existing literature in two important ways:

- our identification procedure depends only on a small set of primitive assumptions on preferences, which is in contrast to much of the previous literatures; and
- our test for social preferences is completely nested in a market for credence goods.

This latter feature might help to alleviate the concern that the results of elicitation procedures based on dictator games are not robust and not easy to extend to other important economic situations.

## 5 Conclusions

This study argues that heterogeneity in social preferences provides an explanation for both, why credence goods markets with verifiability fail to reach efficient outcomes and why markets without verifiability perform considerably better than predicted by standard theory. Key to our argument are the following two observations: first, the standard prediction for markets without verifiability is non‐robust against the presence of agents with pro‐social other‐regarding preferences. Second, the standard solution to the credence goods problem for the case where the quality of the goods is verifiable – equal‐mark‐up prices – is non‐robust against the presence of agents with anti‐social other‐regarding preferences.

To provide support for our explanation, we design a test that allows for a clean discrimination between different preference types from the provision behaviour in an experimental market for credence goods. An important feature of our experimental design is that the discrimination does not depend on any specific assumptions on the form of the utility function of the expert. The experimental design rather directly tests the key characteristics of different variants of social preferences that have been discussed in the economics literature. A second important design feature is that our test for social preferences is completely nested in a market for credence goods.

Important conclusions for credence goods markets and, more generally, for markets with asymmetric information can be drawn from our experimental results. Specifically, we have found in an implementation of our test that less than a quarter of the experimental sellers behave according to the standard assumption on preferences (that all agents are rational own‐money maximisers who behave as desired if held indifferent in own‐money terms). The rest behave either in line with other forms of selfish or in accordance with different variants of non‐selfish other‐regarding preferences. An immediate implication is that institutional design based on the standard assumption of lexicographically maximising agents yields bad incentives for some or possibly many agents. Another implication of our experimental results is that there are agents that behave appropriately, independent of the institutional design. Taken together these two observations have two important consequences, one for institutional design and the other for agent selection.

### 5.1 Designing the Right Institutions

What is needed for a well‐performing market is not a perfect institution for one type of agent but rather an institution that is robust against the coexistence of different types of agents. Our results indicate that verifiability is not such an institution (nor is a market where verifiability does not apply). By contrast, as Dulleck *et al*. (2011) have shown, ‘liability’ is a quite robust institution in markets for credence goods. ‘Liability’ requires verifiability of ‘outcomes’, while ‘verifiability’ requires only verifiability of ‘inputs’. Thus, securing verifiability of outcomes, where possible, might solve credence goods problems more effectively in some markets.

### 5.2 Selecting the Right Agents

Designing robust institutions might be difficult, especially for markets for credence goods. Imposing liability, for instance, generates other problems or may be impossible to achieve.^16^ As a consequence, selecting the ‘right’ agents for jobs involving experts’ services becomes particularly important. Instead of choosing doctors, mechanics or computer specialists exclusively according to their training, customers or their representatives should also take into consideration the attitudes of these experts towards their customers. Selecting the right agents may also help to solve problems created by uncertainty over input costs: with cost uncertainty standard theory would predict that verifiability cannot solve the problems on credence goods markets – a problem ignored in the formal literature on credence goods thus far. Our results suggest that verifiability can solve this problem if the ‘right’ agents are selected: Efficiency loving experts provide appropriate treatment in a corridor along the equal‐mark‐up line; that is, even if monetary incentives are not perfectly in line. Hence, the crucial task of potential employers or buyers is to identify experts with the right social preferences. Public policy might step in here, for instance, by screening applicants for particular jobs (like in the health care sector, for instance) not only after their performance in entry examinations but also in accordance with their social track record. Since the ‘effort cost’ for performing social activities is arguably lower for more ‘consumer‐friendly’ types, a CV featuring an impressive track record of volunteer work might well act as a screening device.

## Supporting information


**Appendix A.** Social Preference Types.
**Appendix B.** Proof of Lemma 1.
**Appendix C.** Additional Experimental Results.
**Appendix D.** Experimental Instructions for the ‐Exo Treatments.

**Data S1.**

## Notes

1 Economically important credence goods markets include the market for medical care and that for car repair services. For the former the data in the WHO World Health Statistics (2009) – online available at http://www.who.int/whosis/whostat/2009/en/ – indicate that health care expenditures account for approximately 15% of GDP in the US and are still rising. For the latter the online site researchandmarkets.com reports annual revenues of about $90 billion for the US auto repair industry, of which 70% originate from mechanical repair.

2 See Emons (1997, 2001), Pesendorfer and Wolinsky (2003), Alger and Salanié (2006) and Dulleck and Kerschbamer (2009) for research articles on the role of verifiability and Dulleck and Kerschbamer (2006) for a unifying model and a survey of the literature.

3 Typical assumptions made in the economic literature on social preferences are linearity (the ring‐test – employed by Offerman *et al*. (1996) and Brandts *et al*. (2009), among others – is based on the assumption of linear preferences), piecewise linearity (the tests implemented by Cabrales *et al*. (2010), Blanco *et al*. (2011) and Iriberri and Rey‐Biel (2013) are based on a piecewise linear model of social preferences), or specific forms of convexity (Andreoni and Miller (2002) and Fisman *et al*. (2007) check consistency of behaviour with the maximisation of a CES utility function).

4 An exception is Kerschbamer (2015) who develops a test for social preferences that shares many features with the one proposed here. We discuss the relationship further in Section 2.

5 Dulleck *et al*. (2013) address in a similar framework the question whether a seller's price‐posting behaviour is indicative of her intentions regarding provision and charging behaviour. Huck *et al*. (2007, 2010, 2012) have interesting experiments on the effect of prices and opportunities to build up a reputation on the performance of markets for experience (rather than credence) goods.

6 Dulleck *et al*. (2011) have a total of 16 experimental treatments (on the role of liability, verifiability, competition and reputation) of which we discuss only two here (and add two new ones). The main difference in experimental design between the new treatments and those in Dulleck *et al*. (2011) is our reliance on (carefully designed) exogenously given prices for different qualities of the good rather than letting sellers endogenously decide on prices. To emphasise this difference, we refer to the treatments B/N and B/V in Dulleck *et al*. (2011) as treatments N‐Endo and V‐Endo here, while the new treatments have names ending in ‐Exo.

7 See Kerschbamer (2015) for a more detailed discussion of this assumption.

8 The test proposed by Kerschbamer (2015) relies on the somewhat weaker assumption d*U*(*z*,*z*)/d*z* > 0 for all *z* > 0. This assumption only rules out more extreme variants of spite while the condition in the text excludes both extreme variants of altruism and extreme variants of spite.

9 In online Appendix A, we discuss those archetypes and their relation to different variants of social preferences discussed in the literature in some detail. There we also present a Figure that displays typical indifference curves for the five archetypes in $\left(\pi_{s},\pi_{c}\right)$ space. By focusing on the five ‘pure’ types defined in Table 3 we omit preference types that are selfish in one of the two domains and pro‐ or anti‐social in the other. In the experimental section (Section 5), we allow for such hybrid types. In online Appendix A, we explain how this is done.

10 Point Ω and the other price‐vectors indicated by bullet points in Figure 5 are not important for the arguments in this section – we will refer to them in the next section.

11 Table 5 includes data for the equal‐mark‐up price‐vector (4,8). In treatment V‐Endo, where prices are chosen by sellers this price‐vector is observed extremely rarely – specifically in only 17 of the 704 possible cases. In two of the 17 cases, no interaction took place. The overtreatment rate in the remaining cases is 37.5%, which is in the range that is observed in V‐Exo1 and V‐Exo2. The undertreatment rate is zero in V‐Endo under this price‐vector but it is based on only seven cases, which prevents meaningful testing of whether the provision behaviour under equal‐mark‐up price‐vectors depends on whether the price‐vector has been chosen endogenously or determined exogenously.

12 We included all experts who under price‐vector Ω had treated at least one customer needing $q^{0}$ AND at least one customer needing $q^{1}$; 50 of the 56 sellers were included under this rule. From the remaining six sellers, we included those where the data were consistent with exactly one of the social preference types introduced in Section 3.

13 Again, we pool the data from V‐Exo1 and V‐Exo2, because a Fisher exact test reveals that there are no significant differences in the distribution of social preference types between the two treatments (with p > 0.5).

14 Formally, the reason is that the weak SE type is the limit of all kinds of social preference types when the weight on the distributional part of the utility function approaches zero. Note, however, that the limiting behaviour is different for the four non‐SE types!

15 Table C3 in the online Appendix C displays a Table equal to Table 6 but based only on the 45 sellers who respect the monotonicity condition throughout the 16 periods of play.

16 On the one hand, liability requires a form of verifiability of success. Especially in the medical realm success is often impossible or very costly to measure for a court, while still being observed by the consumer. On the other hand, even in cases where success is verifiable strict liability might pose problems. For instance, an insufficiently repaired car may work for some time before it breaks down. To mitigate the undertreatment problem in such a situation the liability needs to cover a longer period. But during this longer period the car may stop working for reasons unrelated to the expert's behaviour. Also, an extended liability period may induce fraudulent behaviour on the side of the customer as he may not put in the required maintenance effort – a problem that has previously been discussed by Taylor (1995).
