# Supplementary material for: How Social Preferences Shape Incentives in (Experimental) Markets for Credence Goods
Source: Econ J (London). 2016 Feb 23;127(600):393–416. doi: 10.1111/ecoj.12284 (PMC5347901; doi:10.1111/ecoj.12284)
Supplement: Supplementary file 1 — Appendix A. Social Preference Types. Appendix B. Proof of Lemma 1. Appendix C. Additional Experimental Results. Appendix D. Experimental Instructions for the ‐Exo Treatments. [file ECOJ-127-393-s001.pdf]

The Economic Journal, Doi: 10.1111/ecoj.12284 © 2015 The Authors. The Economic Journal published by John Wiley & Sons Ltd on behalf of Royal Economic Society. Published by John Wiley & Sons, 9600 Garsington Road, Oxford OX4 2DQ, UK and 350 Main Street, Malden, MA 02148, USA.

Technical Appendix to

HOW SOCIAL PREFERENCES SHAPE INCENTIVES IN (EXPERIMENTAL) MARKETS FOR CREDENCE GOODS

Rudolf Kerschbamer, Matthias Sutter and Uwe Dulleck

Economic Journal, doi: 10.1111/ecoj.12284

Appendix A. Social Preference Types

To keep the discussion in Sections 3 and 4 succinct, we focus in those Sections on the five 'pure' types of social preferences defined in Table 3. Figure A1 displays typical indifference curves for those types in the monetary payoffs space (seller's own pay-off on the horizontal axis and customer's pay-off on the vertical axis).

- A selfish (SE) seller is a homo oeconomicus according to standard theory – she simply maximises her own material pay-off. Thus, since the pay-off of the customer does not affect the seller's utility, the indifference curves of a SE seller in (π_s, π_c) space are vertical.
- An efficiency loving (EL) expert is willing to give up own monetary pay-off to increase the material pay-off of her trading partner if the 'price of giving' is not too high. Thus, the indifference curves of an EL expert in (π_s, π_c) space are downward sloped

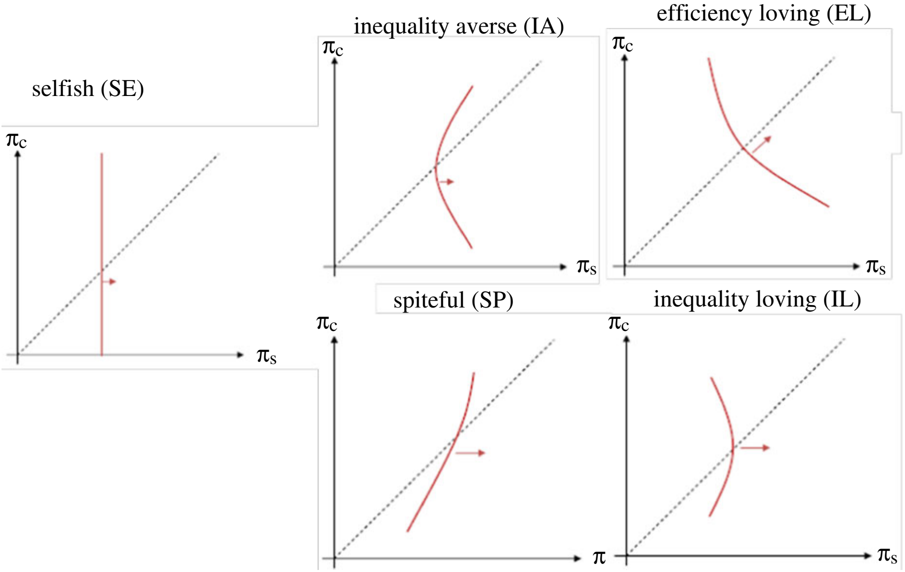

Fig. A1. *Indifference Curves of SE, IA, EL, SP and IL Experts in* (π_s, π_c) *Space*
Notes. A downward sloping segment of an indifference curve implies benevolent (or pro-social) preferences in the respective domain while an upward sloping segment of an indifference curve implies malevolent (or anti-social) preferences in the respective domain.

everywhere. Note that this class subsumes altruists, as modelled by Andreoni and Miller (2002), for instance, as well as surplus maximisers, as discussed by Engelmann and Strobel (2004), among others.

- A spiteful (SP) expert is willing to give up own monetary pay-off to decrease the pay-off of her trading partner if the 'price of taking' is not too high. Thus, the indifference curves of a SP seller in (π_s, π_c) space are upward sloped everywhere. Note that this class includes spiteful experts, as modelled by Levine (1998), for instance; competitive sellers *à la* Charness and Rabin (2002); and experts with concerns for relative income *à la* Duesenberry (1949).
- An inequality averse (IA) expert wants to see the pay-off of her customer increased if she is better off than the customer but she wants to see the customer's pay-off decreased if the opposite is the case. Thus, the indifference curves of an IA expert in (π_s, π_c) space are downward sloped below the equal-monetary-pay-offs line and upward sloped above that line. Note that this class includes inequity or inequality averse agents, as modelled by Fehr and Schmidt (1999), or Bolton and Ockenfels (2000), for instance; agents with egalitarian motives, as discussed by Dawes *et al.* (2007) and by Fehr *et al.* (2008), among others; as well as difference averse agents *à la* Charness and Rabin (2002) and Fisman *et al.* (2007).
- An inequality loving (IL) expert is willing to sacrifice own material pay-off to increase the difference between the pay-offs of the two trading partners. Thus, the indifference curves of an IL expert in (π_s, π_c) space are upward sloped below the equal-monetary-payoffs line and downward sloped above that line. Note that this class includes equity averse agents as discussed, for example by Charness and Rabin (2002) and by Fershtman *et al.* (2012).

The above distinction between five classes of social preferences omits types that are selfish in one of the two domains and pro or anti-social in the other (see Kerschbamer, 2015 for a distinction between nine archetypes of social preferences). In the experimental section (Section 5), we allow for such hybrid types. We do so by extending the definitions of IA and IL as follows:

- We assign a subject to IA if her behaviour is consistent with the combination:

  (*a*) $\partial U/\partial \pi_c \geq 0$ for $\pi_s \geq \pi_c$;
  (*b*) $\partial U/\partial \pi_c \leq 0$ for $\pi_s < \pi_c$; and
  (*c*) at least one of the two derivatives is different from 0.

- And we assign a subject to IL if her behaviour is consistent with the combination:

  (*a*) $\partial U/\partial \pi_c \leq 0$ for $\pi_s \geq \pi_c$;
  (*b*) $\partial U/\partial \pi_c \geq 0$ for $\pi_s < \pi_c$; and
  (*b*) at least one of the two derivatives is different from 0.

Appendix B. Proof of Lemma 1

Proof. First note that providing q^1 yields the equal-material-pay-offs allocation $\pi_s = \pi_c = (v - c^1)/2$, independently of the consumer's need and independently of whether Ω or Ψ is the relevant price-vector. By contrast, the pay-off allocation from providing q^0 depends on both, the consumer's need and the type of contract. Suppose first the consumer needs q^0. Then providing q^0 under Ω yields $\pi_s = (v - c^1)/2$ and $\pi_c = (v - c^1)/2 + (c^1 - c^0)$, while providing q^0 under Ψ yields $\pi_s = (v - c^1)/2 + \varepsilon$ and $\pi_c = (v - c^1)/2 + (c^1 - c^0) - \varepsilon$, where $\varepsilon > 0$ for $p^0{}_\Omega < p^0{}_\Psi$ and $\varepsilon < 0$ for $p^0{}_\Omega > p^0{}_\Psi$. Now, suppose the consumer needs q^1. Then providing q^0 under Ω yields $\pi_s = (v - c^1)/2$ and $\pi_c = (v - c^1)/2 + (c^1 - c^0) - v$, while providing q^0 under Ψ yields $\pi_s = (v - c^1)/2 + \varepsilon$ and $\pi_c = (v - c^1)/2 + (c^1 - c^0) - v - \varepsilon$, where $\varepsilon > 0$ for $p^0{}_\Omega < p^0{}_\Psi$ and $\varepsilon < 0$ for $p^0{}_\Omega > p^0{}_\Psi$. It remains to be shown that $U(\phi + \varepsilon, \chi - \varepsilon)$ is increasing in ε. This follows from $\partial U/\partial \pi_s > \partial U/\partial \pi_c$ for all (ϕ, χ).

© 2015 The Authors.
The Economic Journal published by John Wiley & Sons Ltd on behalf of Royal Economic Society.

Appendix C. Additional Experimental Results

Table C1

Overview of Results in V-Exo1 and V-Exo 2 (all periods)

	V-Exo1	V-Exo2	p-value
Interaction	0.62	0.64	0.29
Undertreatment	0.57	0.49	0.75
Overtreatment	0.44	0.17	0.00
Profit seller	2.28	2.48	0.07
Profit buyer	1.20	1.44	1.00
Number of subjects	64	64	
Matching groups	8	8	

Table C2

Undertreatment (UT) and Overtreatment (OT) Rates Conditional on Price-vectors (all periods)

	UT*	UT*	p-value	OT†	OT†	p-value
$p_{\mathrm{I}}, p_{\mathrm{II}}$	V-Exo1	V-Exo2	(UT)	V-Exo1	V-Exo2	(OT)
(3,8)	0.036	n.a.	–	0.935	n.a.	–
(4,8)	0.237	0.211	0.60	0.409	0.414	0.96
(5,8)	0.588	0.681	0.6	0.040	0.059	0.62
(6,8)	0.657	0.606	0.87	0.077	0.026	0.84
(7,8)	0.588	0.772	0.07	0.056	0.038	0.93

Notes. *Undertreatment: customer needs q^1, but seller provides q^0. †Overtreatment: customer needs q^0, but seller provides q^1.

Table C3

Classification of Individual Behavior in V-Exo (all periods)

Social preference type	Strong	Weak	Total
EL (efficiency loving)	13 (28.9%)	8 (17.8%)	21 (46.7%)
IA (inequality averse)	10 (22.2%)	3 (6.7%)	13 (28.9%)
SP (spiteful)	0 (0%)	2 (4.4%)	2 (4.4%)
IL (inequality loving)	0 (0%)	2 (4.4%)	2 (4.4%)
SE (selfish)	7 (15.6%)	15 (33.3%)	22 (48.9%)

Table C4

Average Profits of Sellers Per Period, Conditional on Preference Type and Conditional on Interaction (based on the classification in Table 6 – see number of observations there)

Social preference type	Strong	Weak	Total
EL (efficiency loving)	2.37	2.45	2.41
IA (inequality averse)	2.22	2.69	2.33
SP (spiteful)	–	2.25	2.25
IL (inequality loving)	–	2.37	2.37
SE (selfish)	2.55	2.45	2.48

© 2015 The Authors.
The Economic Journal published by John Wiley & Sons Ltd on behalf of Royal Economic Society.

Table C5

Average Profits of Sellers Per Period, Conditional on Preference Type and Conditional on Interaction (based on the classification in Table C3 – see number of observations there)

Social preference type	Strong	Weak	Total
EL (efficiency loving)	2.37	2.49	2.42
IA (inequality averse)	2.22	2.69	2.33
SP (spiteful)	–	2.21	2.21
IL (inequality loving)	–	2.37	2.37
SE (selfish)	2.64	2.48	2.53

Appendix D. Experimental Instructions for the -Exo Treatments

INSTRUCTIONS FOR THE EXPERIMENT

Thank you for participating in this experiment. Please do not to talk to any other participant until the experiment is over.

2 Roles and 16 Rounds

This experiment consists of 16 rounds, each of which consists of the same sequence of decisions. This sequence of decisions is explained in detail below.

There are two kinds of roles in this experiment: player *A* and player *B*. At the beginning of the experiment you will be randomly assigned to one of these two roles. On the first screen of the experiment you will see which role you are assigned to. Your role remains the same throughout the experiment.

A player *A* interacts with a player *B*. This pair of players changes for each round. Therefore, you are interacting in every round with a new player (of the other role).

All participants get the same information on the rules of the game, including the costs and payoffs for both players.

Overview of the Sequence of Decisions in a Round

Each round consists of a maximum of two decisions which are made consecutively. Decision 1 is made by player *B* and decision 2 is made by player *A*. In each round, 2 prices will be announced before players make their decisions. These prices are set for a given round. This price setting is referred to in the following as 'Decision 0'.

Short Overview of the Sequence of Decisions in a Round

0. The prices for action I and action II are announced to both players.
1. Player *B* decides whether he/she wants to interact with player *A*. If he/she chooses No, the round ends.

If player *B* chooses to interact then

2. Player *A* (but not player *B*) is informed about the type of player *B*. There are two possible types of player *B*: he/she is of either type I or type II. Player *A* has to choose an action: either action I or action II. He/she then receives the price for the chosen action valid for this round. This price has to be paid by player *B*.

© 2015 The Authors.
The Economic Journal published by John Wiley & Sons Ltd on behalf of Royal Economic Society.

Detailed Illustration of the Decisions and Their Consequences Regarding Pay-offs

Decision 0

In case of an interaction player *A* has to choose between two actions, action I and action II, in Decision 2. Each chosen action causes costs which are as follows:

Action I results in a cost of 2 points (=currency of the experiment) for player *A*.

Action II results in a cost of 6 points for player *A*.

Player *A* receives from player *B* the valid price for the action he/she chooses in Decision 2 if player *B* decides to interact with him/her. At Decision 0 the valid prices for action I and action II for this round are announced to both players.

Decision 1

Player *B* decides whether he/she wants to interact with player *A*.

If he/she wants to do so, then player *A* chooses an action in Decision 2 and he/she receives the valid price for this action from player *B*.

If he/she does not want to interact, then this round ends and both players get a payoff of 1.6 points for this round.

Decision 2

Before Decision 2 is made (in case player *B* chose 'Yes' at Decision 1) a type is randomly assigned to player *B*. Player *B* can be of one of two types: type I or type II. This type is determined a new in each round. With a probability of 50% player *B* is of type I, and with a probability of 50% he/she is of type II. Imagine that a coin is tossed in each round. If, for example the result is 'heads', player *A* is of type I, if it is 'tails' he/she is of type II.

Player *A* gets to know the type of player B before he/she makes Decision 2. Then player *A* chooses an action, either action I or action II, and receives the corresponding price (valid for the respective round).

An action is sufficient under the following conditions:

(*i*) player *B* is a type I player and player *A* chooses either action I or action II;
(*ii*) player *B* is a type II player and player *A* chooses action II.

An action is not sufficient if player *B* is of type II and player *A* chooses action I.

Player *B* receives 10 points, if the action chosen by player *A* is sufficient. Player *B* receives 0 points if the action chosen by player *A* is not sufficient. In both cases player *B* has to pay the valid price for the chosen action.

At no time will player *B* be informed about whether he/she is of type I or a type II in any given round.

Payoffs

If player *B* chooses not to interact in Decision 1 (decision 'No' of player *B*) then both players receive 1.6 points for this round.

Otherwise (decision 'Yes' by player *B*) the payoffs are as follows:

Player *A* receives the price (denoted in points, as announced in Decision 0) for the action chosen in Decision 2, less the cost of this action.

Player *B*'s pay-off depends on whether the Decision 2 of player *A* was sufficient or not.

(*i*) If the action was sufficient, player *B* gets 10 points less the price for the action chosen by player *A* in Decision 2.

© 2015 The Authors.
The Economic Journal published by John Wiley & Sons Ltd on behalf of Royal Economic Society.

(*ii*) If the action was not sufficient, player *B* has to pay the price for the action chosen by player *A* in Decision 2.

At the beginning of the experiment you receive an initial endowment of 6 points. With this endowment you are able to cover losses that might occur in some rounds. Losses can also be compensated by gains in other rounds. If your total pay-off sums up to a loss at the end of the experiment you will have to pay this amount to the supervisor of the experiment. By participating in this experiment you agree to these terms. Please note that there is always a possibility to avoid losses in this experiment.

To calculate the final pay-off the initial endowment and the profits of all rounds are added up. This sum is then converted into cash using the following exchange rate:

1 point = 25 euro-cents

(i.e. 4 points = 1 euro)

References

Andreoni, J. and Miller, J. (2002). 'Giving according to GARP: an experimental test of the consistency of preferences for altruism', *Econometrica*, vol. 70(2), pp. 737–53.

Bolton, G. and Ockenfels, A. (2000). 'ERC: a theory of equity, reciprocity, and competition', *American Economic Review*, vol. 90(1), pp. 166–93.

Charness, G. and Rabin, M. (2002). 'Understanding social preferences with simple tests', *Quarterly Journal of Economics*, vol. 117(3), pp. 817–69.

Dawes, C., Fowler, J., Johnson, T., McElreath, R. and Smirnov, O. (2007). 'Egalitarian motives in humans', *Nature*, vol. 446(7137), pp. 794–6.

Duesenberry, J. (1949). *Income, Saving and the Theory of Consumer Behavior*, Cambridge, MA: Harvard University Press.

Engelmann, D. and Strobel, M. (2004). 'Inequality aversion, efficiency, and maximin preferences in simple distribution experiments', *American Economic Review*, vol. 94(4), pp. 857–69.

Fehr, E. and Schmidt, K. (1999). 'A theory of fairness, competition, and cooperation', *Quarterly Journal of Economics*, vol. 114(3), pp. 817–68.

Fehr, E., Bernhard, H. and Rockenbach, B. (2008). 'Egalitarism in young children', *Nature*, vol. 454(1079), pp. 1079–83.

Fershtman, C., Gneezy, U. and List, J. (2012). 'Equity aversion: social norms and the desire to be ahead', *American Economic Journal: Microeconomics*, vol. 4(4), pp. 131–44.

Fisman, R., Kariv, S. and Markovits, D. (2007). 'Individual preferences for giving', *American Economic Review*, vol. 97(5), pp. 1858–76.

Kerschbamer, R. (2015). 'The geometry of distributional preferences and a non-parametric identification approach: the equality equivalence test', *European Economic Review*, vol. 76(1), pp. 85–103.

Levine, D. (1998). 'Modeling altruism and spitefulness in experiments', *Review of Economic Dynamics*, vol. 1(3), pp. 593–622.

© 2015 The Authors.
The Economic Journal published by John Wiley & Sons Ltd on behalf of Royal Economic Society.
