# Supplementary material for: How Social Preferences Shape Incentives in (Experimental) Markets for Credence Goods
Source: Econ J (London). 2016 Feb 23;127(600):393–416. doi: 10.1111/ecoj.12284 (PMC5347901; doi:10.1111/ecoj.12284)
Supplement: Supplementary file 2 — Data S1. [file ECOJ-127-393-s002.zip › File_5_Supplementary_Material_and_Information.pdf]

Supplementary material and information to

How Social Preferences Shape Incentives in (Experimental) Markets for Credence Goods

Rudolf Kerschbamer, Matthias Sutter, and Uwe Dulleck
(forthcoming in: Economic Journal)

This paper builds on two experimental treatments taken from Dulleck, Kerschbamer and Sutter (2011 in American Economic Review) and two new treatments, called V-Exo1 and V-Exo2.

The two treatments from Dulleck et al. (2011) are denoted **N-Endo** and **V-Endo** in the EJ-paper, but are denoted B/N and B/V in the original paper of Dulleck et al. (2011). To retrieve the data for B/N and B/V, the reader is referred to the AER-webpage

https://www.aeaweb.org/articles.php?doi=10.1257/aer.101.2.526

There, the data set and the supplementary material for replication are available.

The new treatments, **V-Exo1** and **V-Exo2**, were run with zTree by Urs Fischbacher (2007), and the program for running the experiment is included as files

File_6_EXO1_credenceGoods_T3-(V+NL)_Fixpreise_v2.ztt
File_7_EXO2_credenceGoods_T3-(V+NL)_Fixpreise_v2.ztt

The raw data for the six sessions (4 sessions for V-Exo1 with 16 subjects each; 2 sessions for V-Exo2 with 32 subjects each) are included in an Excel-file labelled

File_8_Kerschbamer+Sutter+Dulleck_Exo_Data_EJ.xlsx

In this Excel-file, there is a sheet labelled "Group_averages" where data on the matching-group level (of eight subjects each) is given. We used these matching-group level data for the analysis. Note, however, that the data in the tables of the paper do not simply take averages over matching groups, since the number of observations might be different across matching groups. Therefore, for variables like undertreatment or overtreatment we consider for the numbers in the tables in the paper the number of cases where undertreatment or overtreatment can actually occur. Here note that undertreatment can only occur if the customer needs the high quality, while overtreatment is only an issue if the customer needs the low quality – and these numbers can differ across matching-groups.

See below for a description of the most important variables in the Excel-file.

The **instructions** for the V-Exo-treatments were originally in German, and the original is included as file

File_9_Instruktionen_T3(V)_Fixpreise_v3.pdf

An English translation is included in Part D of the Online Appendix to the paper.

The experimental **design** is carefully described in the paper and is not repeated here. The experiment was run with students at the University of Innsbruck. At the time of running the experiments, the database contained about 3,000 students who had voluntarily registered for taking part in economic experiments. The invitations for registrations were sent once a year to new students who had registered at the University of Innsbruck. When inviting subjects, we excluded all those who had previously participated in credence goods experiments. The invitation came with the option for signing up for specific sessions. Of course, we did not mention any details about the experiment or its different treatments when sending out invitations.

Description of most important variables in the Data-file
File_8_Kerschbamer+Sutter+Dulleck_Exo_Data_EJ.xlsx

Exo_1 = 1 if treatment is V-Exo1, 0 otherwise (is V-Exo2).
Profit = profit per period in points
Type: 1 = Seller, 2 = Buyer
Property: 1 = type 1 for buyer; 2 = type 2 for buyer
Action: 1 = action 1 taken by seller; 2 = action 2 taken by seller
Interaction: 1 = interaction takes place (buyer chooses Yes in decision 1); 0 = no interaction
Sufficient: 1 = action by seller sufficient for buyer; 0 otherwise
price[1]: price for action 1
price[2]: price for action 2
