# Supplementary material for: How Social Preferences Shape Incentives in (Experimental) Markets for Credence Goods
Source: Econ J (London). 2016 Feb 23;127(600):393–416. doi: 10.1111/ecoj.12284 (PMC5347901; doi:10.1111/ecoj.12284)
Supplement: Supplementary file 2 — Data S1. [file ECOJ-127-393-s002.zip › File_9_Instruktionen_T3(V)_Fixpreise_v3.pdf]

ANLEITUNG ZUM EXPERIMENT

Herzlichen Dank für Ihre Teilnahme am Experiment. Bitte sprechen Sie bis zum Ende des Experiments nicht mehr mit anderen Teilnehmern.

2 Rollen und 16 Runden

Dieses Experiment besteht aus **16 Runden**, die jeweils die gleiche Abfolge an Entscheidungen haben. Die Abfolge der Entscheidungen wird unten ausführlich erklärt.

Es gibt im Experiment 2 Rollen: **Spieler A** und **Spieler B**. Zu Beginn des Experiments bekommen Sie eine dieser Rollen zufällig zugelost. Auf dem ersten Bildschirm des Experiments sehen Sie, welche Rolle Sie haben. Diese Rolle bleibt für alle Spielrunden gleich.

Ein Spieler A interagiert immer mit einem Spieler B. Allerdings **wechseln** die Paare nach jeder Runde. D.h. dass Ihnen in jeder Runde zufällig ein Spieler (der anderen Rolle) zugeteilt wird.

Alle Experimentteilnehmer erhalten die gleichen Informationen bezüglich der Regeln des Spiels, inklusive der Kosten und Auszahlungen an beide Spieler.

Überblick über die Entscheidungen in einer Runde

Jede einzelne Runde besteht aus maximal 2 Entscheidungen, die hintereinander getroffen werden. Die Entscheidung 1 wird von Spieler B getroffen; die Entscheidung 2 wird von Spieler A getroffen. In jeder Runde werden 2 verschiedene Preise bekanntgegeben bevor die beiden Spieler ihre Entscheidung zu treffen haben. Diese beiden Preise sind für die gegebene Runde gültig. Diese Preissetzung bezeichnen wir im Folgenden als „Entscheidung 0“.

Ablauf der Entscheidungen einer Runde (kurz gefasst)

0. Es werden die Preise für Aktion I und Aktion II an beide Spieler bekanntgegeben.

1. Spieler B entscheidet, ob er/sie mit Spieler A interagieren möchte. Falls nein, endet diese Runde.

   Falls ja ...

2. Spieler A (aber **nicht** Spieler B) wird über die Eigenschaft von Spieler B informiert. Es gibt zwei mögliche Eigenschaften von Spieler B, er/sie hat entweder die Eigenschaft I oder die Eigenschaft II. Spieler A wählt daraufhin eine Aktion, entweder Aktion I oder Aktion II. Spieler A erhält dann den für die jeweilige Runde gültigen Preis der gewählten Aktion. Dieser Preis ist vom Spieler B zu bezahlen.

Detaillierte Darstellung der Entscheidungen und ihrer Konsequenzen hinsichtlich der Auszahlungen

Entscheidung 0

Wenn eine Interaktion stattfindet, dann wählt **Spieler A** in Entscheidung 2 zwischen zwei Aktionen, einer Aktion I und einer Aktion II. Jede der gewählten Aktionen verursacht Kosten, diese Kosten sind wie folgt:

Die **Aktion I** verursacht **Kosten von 2 Punkten** (= experimentelle Währungseinheit) für Spieler A.
Die **Aktion II** verursacht **Kosten von 6 Punkten** für Spieler A.

Wenn eine Interaktion stattfindet, dann erhält der Spieler A vom Spieler B den in der entsprechenden Runde gültigen Preis für die vom Spieler A in Entscheidung 2 gewählte Aktion. **In Entscheidung 0 werden die für die entsprechende Runde gültigen Preise für Aktion I und Aktion II an beide Spieler bekanntgegeben.**

Entscheidung 1

Spieler B entscheidet, ob er/sie mit Spieler A interagieren möchte.

Falls ja, dann bedeutet das, dass Spieler A in Entscheidung 2 eine Aktion wählen und den dafür gültigen Preis verlangen kann (siehe unten).

Falls nein, dann **endet** diese Runde und beide Spieler erhalten als **Auszahlung für diese Runde 1,6 Punkte**.

Entscheidung 2

Vor der Entscheidung 2 (falls Spieler B in Entscheidung 1 „Ja“ gewählt hat) wird dem Spieler B zufällig eine Eigenschaft zugelost. **Spieler B** kann 2 Eigenschaften haben: **Eigenschaft I** oder **Eigenschaft II**. Die Eigenschaft des Spielers B wird **jede Runde neu** zufällig bestimmt.

Spieler B hat mit der **Wahrscheinlichkeit von 50% die Eigenschaft I** und mit der **Wahrscheinlichkeit von 50% die Eigenschaft II**. Stellen Sie sich in jeder Runde einen Münzwurf vor. Wenn beispielsweise „Kopf“ kommt, dann hätte Spieler B die Eigenschaft I, falls „Zahl“ kommt, hätte er/sie die Eigenschaft II.

Spieler A erfährt *vor* seiner Entscheidung 2 die **Eigenschaft von Spieler B**. Dann wählt Spieler A eine Aktion, entweder Aktion I oder Aktion II, und er/sie erhält dann den dafür in der jeweiligen Runde gültigen Preis.

Eine **Aktion** ist unter folgenden Bedingungen **ausreichend**:

a) Spieler B hat die Eigenschaft I und Spieler A wählt entweder die Aktion I oder die Aktion II.
b) Spieler B hat die Eigenschaft II und Spieler A wählt die Aktion II.

Eine Aktion ist **nicht ausreichend**, wenn Spieler B die Eigenschaft II hat und Spieler A die Aktion I wählt.

Spieler B erhält **10 Punkte**, wenn die von Spieler A gewählte **Aktion ausreichend** ist. **Spieler B** erhält **0 Punkte**, wenn die von Spieler A gewählte **Aktion nicht ausreichend** ist. In beiden Fällen hat der Spieler B den in der entsprechenden Runde gültigen Preis für die vom Spieler A in Entscheidung 2 gewählte Aktion zu bezahlen (siehe unten bei „Auszahlungen“).

Spieler B wird zu **keiner** Zeit auf dem Computerbildschirm darüber informiert, ob er/sie in einer Runde die Eigenschaft I oder die Eigenschaft II hatte.

Auszahlungen
Wenn Spieler B in Entscheidung 1 die Runde beendet (*Entscheidung „**Nein**" von Spieler B*), dann erhalten beide Spieler in dieser Runde **1,6 Punkte**.
Ansonsten (*Entscheidung „Ja" von Spieler B*) sind die Auszahlungen wie folgt:

Spieler A erhält den in der entsprechenden Runde gültigen **Preis** (in Punkten) für die vom Spieler A in Entscheidung 2 gewählte Aktion **abzüglich** der **Kosten** für diese Aktion.

Für **Spieler B** hängt die Auszahlung davon ab, ob die von Spieler A in Entscheidung 2 gewählte Aktion ausreichend war.

a) Die Aktion von Spieler A war ausreichend: **Spieler B** erhält **10 Punkte abzüglich** des gültigen **Preises** für die in Entscheidung 2 gewählte Aktion.
b) Die Aktion von Spieler A war nicht ausreichend: **Spieler B** muss den in der entsprechenden Runde gültigen **Preis** für die in Entscheidung 2 gewählte Aktion bezahlen.

Zu Beginn des Experiments erhalten Sie eine **Anfangsausstattung von 6 Punkten**. Aus dieser Anfangsausstattung können Sie auch mögliche Verluste in einzelnen Runden bezahlen. Verluste in einer Runde sind aber auch durch Gewinne aus anderen Runden ausgleichbar. Sollten Sie am Ende des Experiments in Summe einen Verlust gemacht haben, müssen Sie diesen Verlust an den Experimentleiter bezahlen. Mit Ihrer Teilnahme am Experiment erklären Sie sich mit dieser Bedingung einverstanden. Beachten Sie aber bitte, dass es in diesem Experiment **immer** eine Möglichkeit gibt, Verluste mit Sicherheit zu vermeiden.

Für die Auszahlung werden die Anfangsausstattung und die Gewinne aller Runden zusammengezählt und mit folgendem Umrechnungskurs am Ende des Experiments in bares Geld umgetauscht:

1 Punkt = 25 Euro-Cent
(d.h. 4 Punkte = 1 Euro).
